# Different brain responses to electro-acupuncture and moxibustion treatment in patients with Crohn’s disease

**DOI:** 10.1038/srep36636

**Published:** 2016-11-18

**Authors:** Chunhui Bao, Peng Liu, Huirong Liu, Xiaoming Jin, Vince D. Calhoun, Luyi Wu, Yin Shi, Jianye Zhang, Xiaoqing Zeng, Lili Ma, Wei Qin, Jingzhi Zhang, Xiaoming Liu, Jie Tian, Huangan Wu

**Affiliations:** 1 Key Laboratory of Acupuncture and Immunological Effects, Shanghai University of Traditional Chinese Medicine, Shanghai, 200030, China; 2 Life Sciences Research Center, School of Life Sciences and Technology, Xidian University, Xi’an, Shaanxi, 710071, China; 3 Outpatient Department, Shanghai Research Institute of Acupuncture and Meridian, Shanghai University of Traditional Chinese Medicine, Shanghai, 200030, China; 4 Stark Neurosciences Research Institute, Indiana University School of Medicine, Indianapolis, Indiana, 46202, USA; 5 The Mind Research Network, Albuquerque, NM, 87131, USA; 6 Department of Electrical and Computer Engineering, University of New Mexico, Albuquerque, NM, 87131, USA; 7 Department of Radiology, Shanghai Mental Health Center, Shanghai Jiaotong University School of Medicine, Shanghai, 200030, China; 8 Department of Gastroenterology, Zhongshan Hospital, Fudan University, Shanghai, 200032, China; 9 Endoscopy Center, Zhongshan Hospital, Fudan University, Shanghai, 200032, China

## Abstract

This study aimed to investigate changes in resting state brain activity in remissive Crohn’s Disease (CD) patients after electro-acupuncture or moxibustion treatment. Fifty-two CD patients and 36 healthy subjects were enrolled, and 36 patients were equally and randomly assigned to receive either electro-acupuncture or moxibustion treatment for twelve weeks. We used resting state functional magnetic resonance imaging to assess Regional Homogeneity (ReHo) levels, and Crohn’s Disease Activity Index (CDAI) and Inflammatory Bowel Disease Questionnaire (IBDQ) scores to evaluate disease severity and quality of life. The results show that (i) The ReHo levels in CD patients were significantly increased in cortical but decreased in subcortical areas, and the coupling between them was declined. (ii) Both treatments decreased CDAI, increased IBDQ scores, and normalized the ReHo values of the cortical and subcortical regions. (iii) ReHo changes in multiple cortical regions were significantly correlated with CDAI score decreases. ReHo changes in several subcortical regions in the electro-acupuncture group, and those of several cortical regions in the moxibustion group, were correlated with reduced CDAI. These findings suggest that both treatments improved cortex-subcortical coupling in remissive CD patients, but electro-acupuncture regulated homeostatic afferent processing network, while moxibustion mainly regulated the default mode network of the brain.

Crohn’s disease (CD) is a type of chronic inflammatory bowel disease (IBD). It can affect any area of the gastrointestinal tract, and causes main symptoms including persistent diarrhea, abdominal pain, as well as weight loss^1^. With young adults being the most prevalent demographic group with CD, this disease may impair their quality of life as well as economic well-being^2^,^3^.

Despite the high prevalence rate of Crohn’s disease, the treatment for CD is far from satisfactory^4^,^5^. Pharmacological treatment can induce the remission of CD symptoms, but the side effects of prolonged drug use limit its efficacy as a long-term treatment solution^5^,^6^. As an important part of Traditional Chinese Medicine, acupuncture and moxibustion are used in China and many other countries of the world. They are used for the treatment of a variety of gastrointestinal diseases including IBD^7^,^8^,^9^,^10^,^11^. Although randomized controlled trials^10^,^11^ have demonstrated that patients with CD benefit from acupuncture and moxibustion treatment, the underlying mechanisms of these treatments are unclear.

Recent researches have indicated that regions of the central nervous system involved in the brain-gut axis dysfunction may play an important role in the development and mechanism of IBD, and those abnormal changes in the structure and function of the brain might be directly involved in development of IBD^12^,^13^,^14^,^15^,^16^,^17^. Earlier studies have also confirmed that clinical efficacy of acupuncture relies on the mediation of the central nervous system^18^,^19^,^20^. Electro-acupuncture is a modern method developed from Chinese traditional acupuncture, which relies on manual manipulation of needles. Electro-acupuncture delivers electrical pulses to needles for increasing stimulation to acupuncture points and improving its clinical effect. Moxibustion is a treatment method that uses the heat from moxa burning to stimulate acupuncture points. It has been shown to have effects similar to acupuncture in clinical practice, but is less used and studied. Herb-partition moxibustion is a type of moxibustion that involves igniting a moxa cone on an herbal cake placed on an acupuncture point. Although electro-acupuncture and moxibustion are different in their stimulation, they are believed to generate similar clinical outcomes. Earlier research showed that electro-acupuncture normalizes brain’s functional activity^21^,^22^, but data on the mechanism of moxibustion is scarce. It remains an open question whether these treatment methods induce different responses in the brain and whether their clinical efficacy is linked to the normalization of the brain’s functional activity.

Resting state-functional magnetic resonance imaging (rs-fMRI) reveals brain’s functional activities and organization without external stimulation, which provides a useful tool for studying the mechanisms of acupuncture on the central nervous system. As a new analytical method, regional homogeneity (ReHo) can reflect abnormalities in the synchronization and coordination of spontaneous neuronal activity of brain regions and reveals brain’s pathophysiological changes^23^. Using rs-fMRI technique and ReHo analysis, the present study aimed to compare the effects of electro-acupuncture and moxibustion on brain activities of CD patients in remission. We hypothesized that electro-acupuncture and moxibustion have a regulatory effect on the abnormal brain activities at resting state, evoking similar and different responses from the brain. Specifically, we compared the resting-state brain activities between CD patients in remission and healthy controls (HCs), and investigated the effects of electro-acupuncture and moxibustion on brain activities of CD patients and potential correlations between brain activity changes and clinical outcomes.

## Methods

### Participants

Patients with CD: A total of 52 patients with CD were recruited from the Specialist Outpatient Clinic for Inflammatory Bowel Disease at the Shanghai Research Institute of Acupuncture and Meridian and the Endoscopy Center of the Zhongshan Hospital affiliated with Fudan University. The patients were subjected to systemic and gastrointestinal screening, which included colonoscopy and pathological biopsy. In addition, their C-reactive protein, erythrocyte sedimentation rate and platelet counts were also measured and recorded two weeks prior to fMRI scans. The colon screening was conducted one month prior to the fMRI scans by an experienced endoscopist from the Zhongshan Hospital who was blind to the experimental conditions. The screening was scored based on the Crohn’s disease endoscopic index of severity (CDEIS).

Inclusion criteria: (1) 18–50 years old; (2) more than 6 years of education; (3) remission period lasting 12 months or longer; (4) CDAI score ≤ 150; (5) CDEIS score <3; (6) dominant right hand; (7) no acupuncture treatment in the last three months. Exclusion criteria: (1) C reactive protein >10 mg/L; erythrocyte sedimentation rate >20 mm/h; platelets >300 × 10^9^/L; (2) CD patients who suffered from abdominal fistula, sinus or had CD-related abdominal surgery; (3) patients who received glucocorticoids, anti TNF-α drugs or other biological agents, mental or opioid drugs in the past 3 months; (4) pregnant or lactating women; (5) patients with current or previous history of mental and nervous system disease or head trauma and loss of consciousness; (6) claustrophobic patients, and (7) those with metal implants in the body.

Subjects who received electro-acupuncture or moxibustion treatment also needed to exclude the one who taking immunosuppressive drugs in the past 3 months, because immunosuppressive drugs are suggested to against the effect of acupuncture and moxibustion treatment, most acupuncture studies in the treatment of IBD always rule out such patients^8^,^10^,^11^. Twelve patients excluded for taking azathioprine, and 4 cases refused to receive acupuncture or moxibustion treatment, only accept fMRI scan once. Finally, 36 subjects were enrolled to receive acupuncture or moxibustion intervention. There are no patients had smoking habit.

HCs: 36 right-handed, healthy volunteers were recruited via a newspaper advertisement by the Shanghai University of Traditional Chinese Medicine. They did not receive any medications, and had no gastrointestinal symptoms or had negative test results on colon assessments done within the preceding year.

All participants were evaluated by an experienced gastrointestinal expert from the Zhongshan Hospital of Fudan University. To rule out anyone with psychiatric disorders, mental examinations were conducted by a specialist from the Shanghai Mental Health Center, based on a structured psychiatric interview from the Diagnostic and Statistical Manual of Mental Disorders, 4^th^ edition (DSM-IV).

Two weeks after the baseline period, 36 patients who were willing to receive acupuncture and moxibustion treatment were equally and randomly assigned to either the electro-acupuncture group (EA group) or the moxibustion group (Mox group). The process of random assignment was blind, serially-coded, and confidential. The different groups of CD patients were assigned to different isolated rooms for treatment and were blind to the setup of the study.

The present study complies with the Declaration of Helsinki (Edinburgh version, 2000). The study was approved by the Institutional Review Board of the Yueyang Hospital of Integrated Chinese and Western Medicine affiliated with the Shanghai University of Traditional Chinese Medicine. All study participants signed informed consent forms. The present research has been registered in the Clinical Trials database (NCT01696838, https://clinicaltrials.gov/) on September 25th, 2012.

### Electro-acupuncture and moxibustion treatments

**Figure 1:**
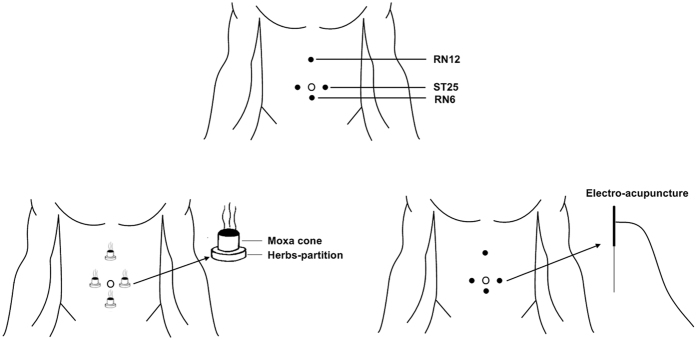
Schematic diagrams show the methods of electro-acupuncture and moxibustion treatments and the locations of acupoints used in this study. The acupoints were ST25 (*Tianshu*), RN6 (*Qihai*) and RN12 (*Zhongwan*).

Each group received treatment three times a week for 12 weeks, totaling up to 36 treatment sessions. The acupuncture points for both groups were chosen in reference to prior research^10^, typical for the treatment of gastrointestinal diseases: ST25 (both sides), CV6 andRN12 (Fig. 1).

#### Electro-acupuncture group

After conventional disinfection with iodine and alcohol, 0.30 × 40 mm needles (Hwato, Suzhou, China) were inserted 20–25 mm beneath the skin and then connected to a HANS electro-acupuncture apparatus (Model LH100A TENS, Nanjing, China). The two conducting ends of the electro-needles were respectively connected to the ST25 and CV6 at the left-hand side, as well as the ST25 and RN12 at the right-hand side. Pulsed stimulus was delivered at a rate of 2/100 Hz and intensity of 1–2 mA for 30 minutes.

#### Moxibustion group

*Radix aconiti preparata, Coptis chinesis, Aucklandia Lappa, Carthamus tinctorius, Salvia miltiorrhiza* and *Angelica sinensis* were ground into fine power and passed through a 100 mesh sieve. The resulting 2.8 g medicinal powder was dissolved in lukewarm water with malt sugar to form a paste. With the use of a mold, the mixture was shaped into an herbal cake with a 23 mm diameter and 5 mm thickness. Moxa cone, with a height of 16 mm and a diameter of 18 mm (“Han Medicine”, Nanyang, China), was placed on top of the herbal cake and ignited for herbs-partitioned moxibustion on each acupuncture point every time.

The dosage remained constant for CD patients who were also receiving mesalazine treatment.

### Outcome measurement

The Crohn’s Disease Activity Index (CDAI)^24^ served as the indication of primary treatment efficacy. CDAI is validated as a measure of CD and treatment efficacy. CDAI < 150 was classified as the remission stage while >150 was classified as the active stage. Participants were assessed at two time points at day 0 and twelve weeks after enrollment in the study.

The Inflammatory Bowel Disease Questionnaire (IBDQ)^25^ was used to assess the quality of life of CD patients. The IBDQ has been validated as an effective measure of health-related quality of life (HRQoL) for adult IBD patients. It consists of 32 questions, each on a 7-point Likert scale, with a total score between 32 and 224. A higher score on the IBDQ indicates a better quality of life. Participants were also assessed at two time points, at day 0 and twelve weeks after enrollment in the study.

### Resting-state fMRI scan

The fMRI data were acquired in an interleaved, multi-slice mode using a SIEMENS TRIO 3T clinical scanner (Magnetom Verio, Siemens, Germany) in the Department of Radiology at the Shanghai Mental Health Center. During the fMRI examination, all patients and HCs were instructed to relax with their eyes closed but not to fall asleep and not to think of anything in particular. They were placed in supine position with the head snugly fixed by a custom-built head holder to minimize head motion, and a pair of foam pads to minimize noise. Functional images were collected with an echo-planar imaging sequence (TR/TE: 2000 ms/30 ms, flip angle = 90°, field of view: 240 mm × 240 mm, matrix size: 240 × 240, in-plane resolution: 1 mm × 1 mm, slice thickness was 5 mm with no gaps, 32 slices). Using a simple questionnaire after the scan, we confirmed that no participants fell asleep during data acquisition. All participants completed the image acquisition procedure.

### Imaging data processing

All of the fMRI data included the 6 min-resting data from HCs and the 6 min-resting data from patients at baseline, as well as the 6 min-resting data from patients at the end of electro-acupuncture or moxibustion treatment. All imaging data were preprocessed using the same procedure.

The resting-state fMRI data were pre-processed using the SPM8 software package (http://www.fil.ion.ucl.ac.uk/spm/software/spm8). The first five images of each functional time series were discarded for longitudinal magnetization to reach the equilibrium. All slices of the remaining images were processed by slice-timing adjustment, and realigned to the first volume. Then, the time series of images of each subject were motion-corrected; the head motion parameters were obtained by estimating six parameters capturing translation in each direction and angular rotation, relative to the first volume. The data set in which the translation or rotation parameters exceeded 1.5 mm or 1.5 degrees of rotation were discarded. The realigned functional images were then spatially normalized to the Montreal Neurologic Institute (MNI) space, using the normalization parameters estimated by the T1 structural image unified segmentation, re-sampled to 3 mm voxels. In order to conserve the fine-grained local pattern and to avoid artificial connections, smoothing was not applied to normalized data. Several sources of spurious variance, including estimated motion parameters, linear drift, and average blood oxygen level-dependent (BOLD) signals in ventricular and white matter regions, were removed from the data using linear regression. Temporal bandpass filtrating (0.01–0.08 Hz) was performed to reduce the effect of low-frequency drifts and high-frequency noise^26^. All of these procedures were performed using the DPARSF software (http://www.restfmri.net/forum/DPARSF)^27^.

### ReHo analysis

Individual ReHo maps were generated by calculating the Kendall’s coefficient of concordance (KCC)^28^, and were used to measure the correlation of the time series of a given voxel with the time series of its 26 nearest neighbors, within a gray matter mask in a voxel-wise manner using the REST software (http://restfmri.net/forum/index.php)^27^. When the center cube was on the edge of the gray matter mask, we only calculated ReHo for a voxel if all of remaining nearby voxels were within the gray matter mask. For each participant, the KCC map was normalized by dividing the KCC in each voxel by the mean KCC of the total gray matter using DPARSF software.

### Statistical analysis

#### Clinical variables

Statistical analyses were performed by two blind evaluators using SPSS 16.0 (SPSS Inc., Chicago, IL, USA). The data analysis was based on an intention-to-treat population. Two independent samples *t*-test was used for normally distributed continuous variables. Nonparametric tests (Kruskal-Wallis test) were used for between-group comparisons of abnormally distributed continuous variables. Pearson’s χ^2^ test or Fisher’s exact test was used for between-group comparisons of categorical variables. All *P* values were two-sided and *P* < 0.05 was considered statistically significant.

#### Imaging data

Analysis between the CD patients and HCs: at the second level of analysis, a two-sample test was initially applied to investigate the differences in patients compared to the whole brain ReHo values of HCs, with age, weight and gender as covariates. The statistical threshold was set at *P* < 0.05 (false discovery rate (FDR) corrected) and the cluster size exceeded five.

Analysis of electro-acupuncture and moxibustion treatment: paired *t*-test was then used to compare the whole brain ReHo values before and after electro-acupuncture or moxibustion treatment in different groups. The statistical threshold was also set at *P* < 0.05 (FDR corrected), and the cluster size exceeded five. Age, weight and gender were treated as insignificant covariates.

Based on the brain regions showing differences in ReHo values after electro-acupuncture or moxibustion treatment in the paired t-test, regions of interest (ROIs) were selected from the sets of voxels within 6-mm spheres with centers at the peak of the clusters. Pearson’s correlation analysis was applied to detect the relationship between the mean change in ReHo values (post-treatment minus baseline) in each ROI and the changes in CDAI scores for CD patients with age, gender and weight as covariates. The significance level was set at *P* < 0.05. Bonferroni corrected for multiple comparisons.

All ReHo-related maps were superimposed on a template provided by the MRIcroN software (http://www.cabiatl.com/mricro/) for display and brain regions showing differences were presented in MNI coordinates.

## Results

### Differences between CD patients and HCs

#### Clinical variables

Clinical and demographic characteristics of all study subjects are shown in Table 1. The demographic characteristics of CD patients and HCs, including gender, age, height and weight, were not significantly different (*P* > 0.05) (Table 1).

#### Increased ReHo value in CD

**Figure 2:**
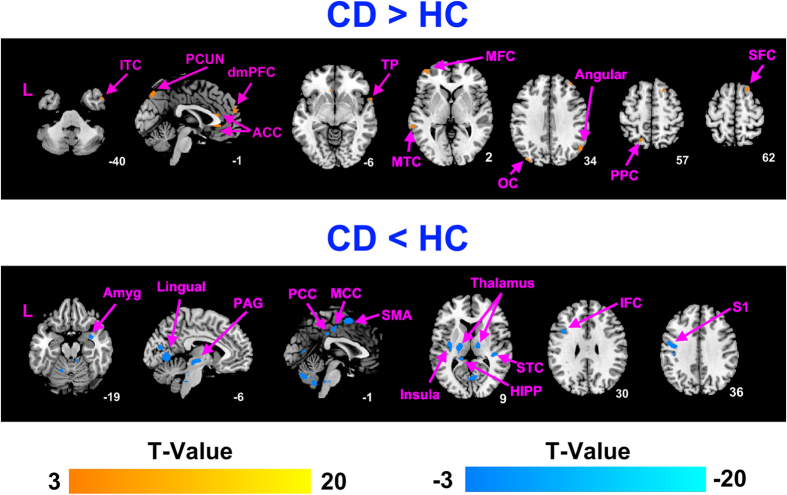
Significant differences in ReHo values between CD patients and HCs. (**a**) The CD patients exhibited significantly increased ReHo values compared to the HCs. (**b**) The CD patients exhibited significantly decreased ReHo values compared to the HCs. *P* < 0.05, false discovery rate was corrected for multiple comparisons with age, gender and weight as covariates. ACC, anterior cingulate cortex; Amyg, amygdala; dmPFC, dorsomedial prefrontal cortex; HIPP, hippocampal cortex; IFC, inferior frontal cortex; ITC, inferior temporal cortex; MCC, middle cingulate cortex; MFC, middle frontal cortex; MTC, middle temporal cortex; OC, occipital cortex; PAG, periaqueductal gray; PCC, posterior cingulate cortex; PCUN, precuneus; PPC, posterior parietal cortex; S1, primary somatosensory cortex; SFC, superior frontal cortex; SMA, supplementary motor area; STC, superior temporal cortex; TP, temporal pole.

In comparison with the HCs, the ReHo values of CD patients were significantly greater in the bilateral ACC, superior frontal medial cortex, middle frontal cortex, superior temporal pole, precuneus, right superior frontal cortex, inferior temporal cortex, angular gyrus, left middle temporal cortex, superior parietal cortex and middle occipital cortex (*P* < 0.05, corrected) (Table 2, Fig. 2a).

#### Decreased ReHo value in CD

In comparison with the HCs, the ReHo values of CD patients were significantly lower in the bilateral thalamus, insula, MCC, PCC, lingual gyrus, cerebellum, PAG, brainstem, left HIPP, SMA, postcentral gyrus, inferior frontal operculum cortex, right amygdala and superior temporal cortex (*P* < 0.05, corrected) (Table 2, Fig. 2b).

### Differences after electro-acupuncture and moxibustion treatment

All 18 patients of the moxibustion group underwent fMRI scans after treatment, which returned useful results. In the electro-acupuncture group, one patient demonstrated head movements during the scan, while another was unable to complete the scans due to schedule conflicts. Ultimately, the fMRI data of the remaining 16 patients were included and analyzed.

#### Clinical variables

The electro-acupuncture and moxibustion groups exhibited no significant differences in age, gender, height, weight, disease course, combination medication as well as CDAI and IBDQ scores (*P* > 0.05) (Table 3).

The CDAI scores of the two groups were significantly lower after treatment (*P* < 0.001, *P* < 0.001), while the IBDQ scores increased significantly (*P* < 0.01, *P* < 0.01). The two treatment groups had no significant difference (from baseline to post-treatment) in the CDAI and IBDQ scores (*P* > 0.05, *P* > 0.05). The reduced CDAI scores and elevated the IBDQ scores in patients of both electro-acupuncture and moxibustion groups suggest that both treatments improved the disease condition and quality of life (Table 4).

#### Changes in ReHo value after electro-acupuncture and moxibustion treatment

In the electro-acupuncture group, there were significant increases in the ReHo values in the bilateral MCC, thalamus, the HIPP, the inferior frontal cortex, the precentral cortex, the paracentral lobule and the superior temporal cortex, the left paraHIPP, the pallidum and cerebellum, the right insula, the putamen, the postcentral cortex, the SMA, the lingual cortex and the brainstem. There were significant decreases in the ReHo values in the bilateral ACC, the orbital frontal cortex, the inferior temporal cortex, the temporal pole and the middle occipital cortex, the left superior parietal cortex, the angular cortex and the cuneus, the right middle frontal cortex and the caudate nucleus (*P* < 0.05, FDR corrected) (Table 5).

In the moxibustion group, significant increases in the ReHo value were observed after treatment in the bilateral MCC, pallidum, putamen, inferior frontal cortex, SMA, middle occipital cortex, cerebellum and brainstem, left PCC, paraHIPP/HIPP, paracentral lobule, angular cortex and fusiform cortex, right insula, precentral cortex and postcentral cortex. Significant decreases in the ReHo value were also detected in the bilateral middle temporal cortex and lingual cortex, left superior medial frontal cortex, inferior temporal cortex and temporal pole, right orbital frontal cortex, middle frontal cortex and the caudate nucleus (*P* < 0.05, FDR corrected) (Table 5).

#### Correlation analysis of brain responses and clinical variables

**Figure 3:**
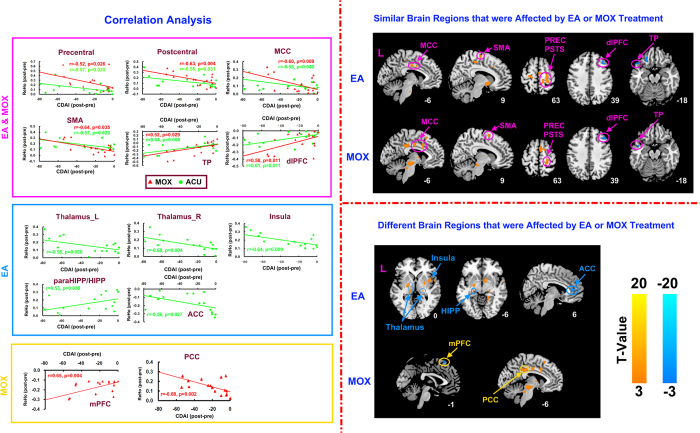
Similar and different brain regions that were affected by EA or moxibuston treatment. (**a**) Correlation analyses between brain responses and CDAI scores. EA&MOX graphs show correlations between changes in ReHo values of different brain regions with common response in EA and moxibustion groups and decreases in CDAI score. EA graphs show correlations between changes in ReHo values of different brain regions in the EA group and a decrease in CDAI score. MOX graphs show correlations between changes in ReHo values of different brain regions in the moxibustion group and a decrease in CDAI score. (**b**) Similar brain regions that were affected by EA or moxibustion treatment. (**c**) Different brain regions that were affected by EA or moxibustion treatment. ACC, anterior cingulate cortex; dlPFC, dorsolateral prefrontal cortex; EA, electro-acupuncture; HIPP, hippocampal cortex; L, left; MCC, middle cingulate cortex; MOX, moxibustion; mPFC, medial prefrontal cortex; PCC, posterior cingulate cortex; PREC, precentral cortex; PSTS, postcentral cortex; SMA, supplementary motor area; TP, temporal pole.

In the electro-acupuncture group, the decrease in the CDAI score was significantly positively correlated with the decreases in the ReHo value in the ACC, the dorsolateral prefrontal cortex (dlPFC) and the temporal pole, and was negatively correlated with the increases in the ReHo value in the MCC, the thalamus, insula, the HIPP, the precentral and the postcentral cortex and SMA after treatment (*P* < 0.05, Bonferroni corrected) (Fig. 3a).

In the moxibustion group, the decrease in the CDAI score was significantly positively correlated with the decreases in the ReHo value in the dlPFC, the dorsomedial prefrontal cortex (dmPFC) and the temporal pole, and was negatively correlated with the increases in the ReHo value in the MCC, the PCC, the precentral and postcentral cortex and the SMA after treatment (*P* < 0.05, Bonferroni corrected) (Fig. 3a).

#### Similar and different responses of brain regions after electro-acupuncture and moxibustion treatments

Electro-acupuncture and moxibustion caused similar activities of left MCC, SMA, right precental cortex, postcentral cortex, dlPFC and temporal pole (Fig. 3b). Additionally, electro-acupuncture caused activities of bilateral thalamus, left HIPP and right insula and ACC, while moxibustion caused activities of left mPFC and PCC (Fig. 3c).

## Discussion

In this study, we examined and compared, for the first time, the effects of electro-acupuncture and moxibustion treatments in regulating brain functional activities in CD patients at resting state. We found that ReHo levels in various brain regions were significantly increased or decreased in CD patients in comparison with HCs, and that the abnormal ReHo values in multiple brain regions were reversed after electro-acupuncture or moxibustion treatments. We also showed that electro-acupuncture and moxibustion treatments have comparable clinical efficacy on improving symptoms of CD, but their effects on functional brain activity includes common and unique brain regions.

### Differences in brain activities in the resting state between CD patients and HCs

In comparison with HCs, the patients with CD exhibited abnormal fluctuations in the ReHo levels in different brain regions. These brain regions were involved in pain, emotion, cognition, attention, as well as homeostasis. The insula, ACC and thalamus are important components of the homeostatic afferent processing network, which is consistently activated in response to homeostatic afferent fiber activation from non-painful and painful visceral and somatic stimuli, as well as emotional stimuli^29^. Abnormal ReHo fluctuations in these brain regions in CD patients indicate declined self-regulatory functions to various stimuli and disruption of homeostasis. The PCC, mPFC, precuneus, angular, amygdale and superior temporal cortex are critical components of the default-mode network (DMN), a system that is involved in self-referential processing and shows greater activation during rest than cognitively demanding tasks^30^,^31^. The detected increase or decrease in ReHo levels in these regions indicate that CD patients at resting state may show abnormalities in neuronal spontaneous activities of the DMN, and be dysfunctional in detecting internal and external environment, situational memory as well as sustained awareness. In addition, the ReHo level in the cortex was significantly increased, while that in the subcortical region was remarkably decreased. The coupling of the cortical and subcortical regions was also declined in patients with CD, leading to abnormal functional separation in signal transmission between them. These findings indicated that patients with CD at resting state likely have abnormalities in synchronization and compatibility in functional activities of brain neurons. Consistently, our earlier study^13^ has demonstrated abnormities in the gray matter structures of the insula, ACC, thalamus, amygdala, mPFC, precuneus, superior temporal cortex and other brain areas of CD patients. In these patients, brain regions with increased gray matter volumes were mainly located in the subcortical regions, cerebellum, and brain stem, while those with decreased gray matter volumes were mainly located in the cerebral cortex. These results also indicate decreased coupling between the cortex and subcortical regions and were consistent with the imaging results in this study. A previous study also showed that CD patients have abnormal neural activity in the amygdala, hippocampus, insula, putamen, cerebellar regions when performing stress-evoking tasks, suggesting altered habituation to stress in these patients^32^. Structurally, CD patients were found to exhibit decreased GM volumes in the frontal and the anterior midcingulate cortex^17^. These findings indicate that there are abnormalities in both structure and functional activities in the brain of CD patients. Dysregulation of homeostatic afferent processing network and DMN, as well as declined cortex-subcortical region coupling, may be critical hallmarks of local brain activities in CD patients at resting state.

### Similarities and differences in the resting-state brain activity between electro-acupuncture and moxibustion treatments

After 12 weeks of treatment, the patients in both the EA and Mox groups demonstrated significant clinical improvement and the abnormal ReHo levels in multiple brain regions were reversed. Although these two methods had similar clinical efficacy and affected several similar brain regions, each of them also affected different brain regions. Brain regions that both electro-acupuncture and moxibustion treatment significantly reduced the CDAI scores and increased the IBDQ scores in patients with CD, suggesting that these methods improve clinical symptoms as well as the quality of life with comparable clinical efficacy. In consistent with this result, our earlier study^33^ also indicated that electro-acupuncture and moxibustion improve the score of the Visual Analogue Scale for Irritable Bowel Syndrome (primary outcomes) in irritable bowel syndrome patients, indicating beneficial regulatory effects of electro-acupuncture and moxibustion on gastrointestinal functions.

In addition, the abnormal ReHo levels in multiple brain regions were corrected after receiving electro-acupuncture and moxibustion treatments. Correlation analysis of efficacy showed that these two treatments resulted in similar activation in certain brain regions and different activation in other regions. Specifically, both treatment methods had similar effect on the MCC and TP of subcortical areas, as well as the precentral cortex, postcentral cortex, SMA and dlPFC of the cerebral cortex. Thus, electro-acupuncture and moxibustion may regulate functional activities in these brain regions, improve the declined cortex-subcortical region coupling, as well as synchronization and compatibility of neuronal spontaneous activities.

Electro-acupuncture and moxibustion also affected different brain regions. Electro-acupuncture significantly affected the insula, ACC, thalamus, and HIPP. These brain regions are closely associated with homeostatic afferent processing. The insula, ACC, and thalamus are recognized as critical regions of the brain-gut axis interaction, and play an important role in processing and regulating pain, emotional visceral sense, as well as homeostasis^34^,^35^. Moreover, the insula and ACC are related to motivations and feelings associated with changes in body’s physiological condition and with the autonomic response and behaviors that occur in optimal balance restoration^29^,^36^. The HIPP can regulate immune response via hypothalamus-pituitary gland-adrenal axis and is closely related to psychoneuroimmunological regulation^37^. In this study, the improvements of CDAI scores of CD patients by electro-acupuncture were significantly correlated with the changes in ReHo level in these brain regions, indicating that clinical efficacy of electro-acupuncture may be medicated by regulating homeostatic emotions, immune function, and the body’s homeostasis. These findings are consistent with earlier studies on the treatment of functional dyspepsia with electro-acupuncture^38^.

We found that the mPFC and PCC are closely related to the clinical efficacy of moxibustion. As important components of the DMN, mPFC and PCC are involved in self-referencing processes^30^,^39^. The DMN has been shown to be deactivated during several different types of meditation, such as concentration, loving-kindness, and during the state of diminished awareness in experienced meditators^40^. A recent study indicated that acupuncture stimulation can deactivate the DMN, which may be derived from enhanced bodily attention to certain parts of the body, suggesting that enhanced bodily attention around the acupoints is significant in the brain’s response to acupuncture stimulation^41^. In this study, we found that the reduced CDAI score due to moxibustion treatment was significantly correlated with ReHo level changes in DMN including mPFC and PCC, indicating that the clinical efficacy of moxibustion may lie in its regulation of DMN and enhanced bodily attention. Patients receiving moxibustion usually experience gratification and warmth, and this stimulation is regarded as a critical factor of its efficacy^42^,^43^. Therefore, we propose that moxibustion regulates the DMN, particularly mPFC and PCC, by making the CD patients more aware of their bodies and feelings in the abdomen. In our earlier studies, we also found that moxibustion suppressed abnormal PFC activation in patients with chronic visceral pain^44^. Therefore, we believe that the effect of moxibustion on CD is partially mediated by its regulation of DMN and enhancing bodily attention on the abdomen region.

One limitation of this study is the lack of a placebo-controlled group. Our placebo-controlled protocol was denied by the Institutional Review Board due to likely progression and recurrence of CD, even in patients in remission. Therefore, we could not rule out non-specific effects of electro-acupuncture and moxibustion. However, this issue may be addressed in future research, such as by reducing the period of electro-acupuncture and moxibustion interventions.

In conclusion, we compared the effects of electro-acupuncture and moxibustion treatments on brain responses in CD patients. We found that both treatments were effective in normalizing the decreased cortical-subcortical coupling of the brain. Our data suggest that regulation of the homeostatic afferent processing network, including the insula, ACC, thalamus, and HIPP, may be the primary pattern of brain response to electro-acupuncture treatment. Regulation of the DMN, including mPFC and PCC, may be the primary pattern of brain response to moxibustion treatment.

## Additional Information

**How to cite this article**: Bao, C. *et al*. Different brain responses to electro-acupuncture and moxibustion treatment in patients with Crohn’s disease. *Sci. Rep.* **6**, 36636; doi: 10.1038/srep36636 (2016).

**Publisher’s note:** Springer Nature remains neutral with regard to jurisdictional claims in published maps and institutional affiliations.

## Figures and Tables

**Table 1:** Demographic and clinical characteristics of CD patients and HCs.

		CD (*n* = 52)	HCs (*n* = 36)	*P* value
Gender (male/female), n		37/15	24/12	0.65
Age (years), mean + SD		30.85 ± 7.60	30.33 ± 5.75	0.73
Height (cm), mean + SD		170.15 ± 6.96	169.47 ± 7.17	0.66
Weight (kg), mean + SD		56.85 ± 9.27	59.58 ± 6.49	0.13
Disease duration (years), mean + SD		6.30 ± 4.07	—	—
CDAI, mean + SD		71.22 ± 42.03	—	—
IBDQ, mean + SD		174.23 ± 26.98	—	—
Platelet^▽^, mean + SD		218.79 ± 44.72	—	—
Erythrocyte sedimentation rate^△^, mean + SD		11.46 ± 5.53	—	—
C, reactive protein^▼^, mean + SD		5.01 ± 5.86	—	—
CDEIS, mean + SD		1.05 ± 0.69	—	—
Montreal classification				
Age at diagnosis	A1	4	—	—
	A2	46	—	—
	A3	2	—	—
Location	L1	10	—	—
	L2	12	—	—
	L3	30	—	—
	L4	0	—	—
Behavior	B1	17	—	—
	B2	3	—	—
	B3	14	—	—
	B1P	5	—	—
	B2P	1	—	—
	B3P	12	—	—
Concomitant medication				
Mesalazine		37	—	—
Azathioprine		12	—	—

^▽^Normal range: 100–300 × 10^9^/L; ^△^Normal range: 0–20 mm/h; ^▼^Normal range: 0–10 mg/L.

**Table 2:** Brain regions exhibiting significant differences in ReHo values between CD patients and HCs.

Regions	Hem	BA	MNI			*t* value	Voxels
			X	Y	Z		
CD < HC							
Insula	L	48	−35	−30	21	−4.77	72
MCC	L	23	−2	−22	46	−3.91	19
	R	23	1	−18	49	−3.25	11
PCC	L	23	−1	−31	37	−3.21	12
Thalamus	L	—	−18	−16	9	−3.77	37
	R	—	12	−16	12	−3.78	42
Amygdala	R	36	33	1	−20	−3.41	5
SMA	L	6/8	−2	6	57	−3.47	34
	R	6	2	4	60	−3.23	29
Inferior frontal cortex	L	48	−37	15	23	−3.33	25
Superior temporal cortex	R	41	37	−30	11	−3.60	15
Hippocampal cortex	L	27	−22	−39	3	−3.22	4
Postcentral gyrus	L	48	−59	−12	21	−3.39	26
Lingual gyrus	L	18	−19	−76	−3	−3.54	52
	R	18	12	−71	−3	−3.14	27
Cerebellum	L	—	−1	−52	−45	−3.44	27
	R	—	2	−52	−45	−3.31	21
PAG	—	—	−6	−24	−7	−3.89	13
Brainstem	—	—	3	−35	−40	−3.09	28
CD > HC							
ACC	L	11	−3	33	−3	3.19	11
	R	24	2	30	14	3.32	14
Middle frontal cortex	L	46	−41	50	18	3.28	20
	R	46	36	44	29	3.13	12
Superior frontal medial cortex	L	10	−3	64	24	3.16	45
	R	10	2	60	24	3.19	69
Superior parietal cortex	L	7	−18	−63	57	3.02	17
Precuneus	L	7	−1	−73	49	3.14	15
	R	7	2	−72	48	4.12	30
Angular gyrus	R	39	55	−63	29	3.26	26
Middle occipital cortex	L	19	−34	−81	40	3.87	15

*P* < 0.05, false discovery rate corrected for multiple comparisons.

ACC, anterior cingulate cortex; BA, Brodmann area; Hem, hemisphere; L, left; MCC, middle cingulate cortex; MNI, Montreal Neurologic Institute; PAG, periaqueductal gray; PCC, posterior cingulate cortex; R, right; SMA, supplementary motor area.

**Table 3:** Demographic and clinical characteristics of CD patients in each group.

	Electro-acupuncture group (*n* = 18)	Moxibustion group (*n* = 18)	Statistical value	*P* value
Age (years), mean + SD	31.61 ± 5.00	27.50 ± 8.08	*t* = 1.836	0.08
Gender (male/female), n	13/5	11/7	*X*^*2*^ = 0.500	0.48
Concomitant medication (yes/no), n	16/2	17/1	*X^2^* = 0.364	0.55
Height (cm), mean + SD	170.67 ± 7.99	170.56 ± 6.75	*t* = 0.045	0.97
Weight (kg), mean + SD	58.08 ± 8.98	56.39 ± 9.65	*t* = 0.545	0.59
Disease duration (years), mean + SD	6.00 ± 4.58	5.14 ± 3.29	*t* = 0.648	0.52
CDAI	76.17 ± 40.11	71.78 ± 42.93	*t* = 0.317	0.75
IBDQ	170.83 ± 22.72	174.78 ± 33.10	*t* = −0.417	0.68

**Table 4:** Clinical outcome measurements.

Items	Electro-acupuncture group (n = 16)	Moxibustion group (n = 18)
CDAI		
Baseline, mean ± SD	76.17 ± 40.11	71.78 ± 42.93
Post-treatment, mean ± SD	48.06 ± 30.50*	40.56 ± 28.53*
Changes from baseline to post-treatment	−28.11 ± 29.81	−31.22 ± 26.14
Statistical value	0.333	
*P* value	0.74	
IBDQ		
Baseline, mean ± SD	170.83 ± 22.72	174.78 ± 33.10
Post-treatment, mean ± SD	188.11 ± 19.54*	190.78 ± 21.81*
Changes from baseline to post-treatment	17.28 ± 19.50	16.00 ± 18.13
Statistical value	0.204	
*P* value	0.84	

Compared with baseline, ^*^P < 0.01.

**Table 5:** Brain regions with significant differences in ReHo values in CD patients after electro-acupuncture and moxibustion treatment (post-treatment minus the baseline).

Electro-acupuncture group								Moxibustion group					
Regions	Hem	BA	MNI			t value	Voxels	BA	MNI			t value	Voxels
			x	y	z				x	y	z		
Inferior frontal cortex	L	45	−46	33	12	3.35	27	48	−55	12	6	3.58	31
	R	48	56	16	12	3.31	21	48	53	12	9	3.14	20
Orbital frontal cortex	L	11	−21	36	−21	−3.90	12	—	—	—	—	—	—
	R	47	34	42	−9	−3.39	12	11	10	34	−13	−3.69	15
Middle frontal cortex	R	46	37	33	39	−3.59	20	46	45	28	39	−4.48	17
Superior medial frontal cortex	L	—	—	—	—	—	—	8	−1	27	63	−3.47	12
Precentral cortex	L	6	−36	−10	45	3.89	23	—	—	—	—	—	—
	R	6	28	−21	63	3.82	20	6	48	4	36	3.39	18
Postcentral cortex	R	3	28	−36	63	5.07	30	3	21	−36	63	3.35	22
Paracentral lobule	L	4	−12	−28	56	4.87	34	6	−12	−17	66	3.12	17
	R	5	9	−29	51	3.14	11	—	—	—	—	—	—
Superior parietal cortex	L	7	−26	−60	69	−3.85	9	—	—	—	—	—	—
Angular cortex	L	39	−49	−62	33	−4.14	7	39	−39	−57	39	3.69	10
SMA	L	—	—	—	—	—	—	8	−6	21	56	3.68	12
	R	3	28	−36	61	4.68	14	6	9	14	51	3.47	17
Inferior temporal cortex	L	21	−55	−3	−28	−3.94	11	20	−41	3	−30	−3.73	7
	R	20	57	−12	−36	−3.47	10	—	—	—	—	—	—
Middle temporal cortex	L	—	—	—	—	—	—	21	−55	10	−21	−3.30	14
	R	—	—	—	—	—	—	37	57	−57	3	−3.67	17
Temporal pole	L	38	−52	23	−18	−3.40	10	21	−45	3	−18	−3.43	9
Superior temporal cortex	L	21	−45	−33	6	4.77	26	—	—	—	—	—	—
	R	38	57	3	−3	3.64	35	—	—	—	—	—	—
Fusiform cortex	L	—	—	—	—	—	—	20	−38	−22	−24	3.36	10
Middle occipital cortex	L	17	−25	−100	0	−3.30	21	19	−27	−85	27	3.01	11
	R	7	33	−60	36	−3.71	18	19	31	−79	36	3.16	17
Cuneus	L	18	−1	−81	33	−3.62	15	—	—	—	—	—	—
Lingual cortex	L	—	—	—	—	—	—	17	1	−77	0	−3.25	12
	R	37	21	−44	−6	3.20	15	17	16	−57	5	−3.08	14
Insula	R	48	42	12	−3	4.57	29	48	37	22	6	3.80	11
ACC	L	32	−4	38	6	−3.10	21	—	—	—	—	—	—
	R	32	6	42	8	−5.22	27	—	—	—	—	—	—
MCC	L	24	−6	−11	39	5.18	17	24	−6	12	39	3.80	32
	R	24	6	4	42	4.29	11	23	6	−24	42	4.49	21
PCC	L	—	—	—	—	—	—	23	−6	−36	34	3.46	34
Thalamus	L	—	−11	−12	2	3.95	45	—	—	—	—	—	—
	R	—	22	−24	0	3.13	21	—	—	—	—	—	—
Parahippocampus/Hippocampus	L	20	−24	−21	−12	3.38	10	—	—	—	—	—	—
Caudate nucleus	R	—	15	21	3	−4.82	13	25	7	13	9	−3.48	11
Pallidum	L	—	−21	−6	−3	5.21	17	—	−13	8	0	4.23	11
	R	—	—	—	—	—	—	—	16	6	0	3.37	23
Putamen	L	—	—	—	—	—	—	—	−21	8	0	4.22	12
	R	—	24	12	6	6.29	13	—	29	6	3	3.98	11
Cerebellum	L	—	−28	−66	−21	3.64	10	—	−30	−59	−54	3.47	19
	R	—	—	—	—	—	—	—	35	−71	−20	4.01	29
Brainstem	—	—	0	−36	−36	3.71	11	—	3	−37	−39	4.18	21

*P* < 0.05, false discovery rate corrected for multiple comparisons.

ACC, anterior cingulate cortex; BA, Brodmann area; Hem, hemisphere; L, left; MCC, middle cingulate cortex; MNI, Montreal Neurologic Institute; PCC, posterior cingulate cortex; R, right; SMA, supplementary motor area.
